# Surgical safety checklist compliance: The clinical audit

**DOI:** 10.1016/j.amsu.2022.104397

**Published:** 2022-08-19

**Authors:** Fahad Gul, Maheen Nazir, Khawar Abbas, Alishba Ashraf Khan, Daniya Shahzad Malick, Hashim Khan, Syed Naqash Haider Kazmi, Arbab Osama Naseem

**Affiliations:** a Department of Surgery, Rawalpindi Medical University, Rawalpindi, Pakistan; b Department of Medicine, Rawalpindi Medical University, Rawalpindi, Pakistan

**Keywords:** Surgical safety checklist, Compliance, Patient safety

## Abstract

**Introduction:**

The surgical safety checklist consists of three components: sign-in, performed before the induction of anesthesia; time-out, performed before skin incision; and sign-out, performed immediately after skin closure or before the patient leaves the operating theatre. This study aims to assess compliance with the World Health Organization (WHO) Surgical Safety Checklist (SSC) and explore the barriers facing in properly implementing the surgical safety checklist in operation theatres of a tertiary care hospital.

**Methodology:**

The observational clinical audit was conducted in Surgical Unit I, Benazir Bhutto Hospital, Rawalpindi, Pakistan. Compliance with the surgical safety checklist was observed before and after the educational intervention. After completion of the clinical audit operating theatre staff was asked about the barriers to compliance with the surgical safety checklist using an interview sheet. Mean, and standard deviation was calculated for quantitative variables, whereas frequencies and percentages were calculated for categorical variables using SPSS version 25.0.

**Results:**

Compliance with all the steps of the surgical safety checklist was improved after an educational intervention, with the highest improvement in compliance (66.7%) observed with the Sign-out step “Count of sponges and needles & instruments complete?” Moreover, filling of the patient board and documentation of procedure in the patient file were also improved. Lack of awareness and training to follow the surgical safety checklist was the commonest barrier to compliance with the surgical safety checklist.

**Conclusion:**

Implementing the surgical safety checklist will not only upgrade the patient safety measures but also integrate teamwork skills and improve the local departmental culture.

## Highlights

• Surgical safety checklist compliance improved after the educational intervention.
• The highest improvement in compliance (66.7%) was observed with the Sign-out step “Count of sponges and needles & instruments complete?”.
• The least improvement in compliance (4.2%) was observed with the Time-out step “Confirm all team members have introduced themselves by name & role?”.
• Lack of awareness and training was the commonest barrier to compliance with the surgical safety checklist.

## 1 Introduction

Surgical care is a crucial component of healthcare service delivery in all healthcare systems. Millions of surgical procedures are performed globally, most of them being undertaken in middle to high-expenditure countries [1]. Considering the high burden of surgical services and complications that can arise during surgical procedures, surgical safety is a global public health concern. A review of the in-hospital adverse events showed that most adverse events were operation-related, and roughly 43% were preventable [2].

In response to the need for surgical safety, WHO launched Safe Surgery Saves Lives in 2006, which highlighted the essential objectives for safe surgical practice, and the WHO surgical safety checklist (SSC) was formulated as an effort to provide surgeons with a concise layout to follow these recommendations that ensure patient safety during surgical procedures [3]. The surgical safety checklist consists of three components: sign-in, performed before the induction of anesthesia; time-out, performed before skin incision; and sign-out, performed immediately after skin closure or before the patient leaves the operating theatre [4]. The checklist employs tactics to improve efficiency in the operating theatre and inculcates teamwork and good communication among the operating staff, all of them essentially working together to make the surgical environment safe for the patient. Implementation of the surgical safety checklist has shown to not only decrease operative morbidity and mortality [3,5] but also foster a patient safety culture and enhances communication [6].

It has been established by many studies that the surgical safety checklist reduces morbidity and mortality, but implementing the checklist in diverse surgical settings throughout the world has been a challenge. A prospective observational study in Colorado revealed suboptimal compliance to the safety checklist and a significant difference in compliance among different surgical specialties [7]. Another survey from Ethiopia revealed a surgical safety checklist compliance rate of 39.7% [8]. A review article highlighted that SSC compliance rates vary significantly among different centers, highly dependent on perceptions, teamwork, and efficient leadership [9].

The role of the SSC in improving patient safety during surgical practice has been well established. Implementing the SSC will require effective leadership, a delegation of responsibilities, and collaboration between the surgical staff [10]. It is imperative to assess compliance to SSC in our hospital setting and explore the barriers to effective compliance, as this will help us implement SSC in our surgical environment and improve patient safety. We conducted this clinical audit to prospectively assess compliance with the SSC and explore the barriers to properly implementing the safety checklist.

## 2 Methodology

This observational clinical audit was conducted in Surgical Unit I, Benazir Bhutto Hospital, Rawalpindi, from 9th March 2022–30th April 2022. Benazir Bhutto Hospital is a tertiary care hospital affiliated with Rawalpindi Medical University, Rawalpindi. Ethical approval for the audit was obtained from the respective surgical department. This clinical audit has been conducted following The Revised Standards for Quality Improvement Reporting Excellence (SQUIRE 2.0) guidelines [11]. Patient data remained solely with the authors, and confidentiality was maintained.

The first cycle of the audit was conducted from 9th to March 31, 2022. Before commencing with data collection, one of the researchers trained the team of data collectors on how the WHO Surgical Safety Checklist was used and ensured their understanding of the auditing tool. A group of four medical students observed and recorded whether different aspects of the/checklist were verbalized and performed during various surgical procedures in four operating theatres of the Surgical Unit I. To minimize bias, operating theatre teams were neither informed nor aware that they were being audited.

The data collection tool was a structured questionnaire with questions related to patient characteristics, namely age, sex, type of surgical procedure, and the WHO Surgical Safety Checklist. Apart from verbalising, some components of the checklist were also assessed for performance (regardless of whether they were read aloud or not). These were marking the surgical site, checking the anesthesia machine, medication, and pulse oximeter at Sign In, displaying essential imaging at Time Out and counting sponges and needles, and specimen labelling at Sign Out.

After the first cycle of the audit was complete, an educational intervention was conducted from 1st to 7^th^ April 2022. This intervention comprised a departmental presentation on the importance of following the surgical safety checklist, results from the first audit cycle (which showed low compliance), and how the checklist is to be followed. Additionally, circulars signed by the head of the department containing instructions to follow the checklist were distributed to all staff members, including doctors, nurses, and operating theatre technicians.

The second cycle was conducted after one week, from 8th to 30^th^ April 2022, using the same data collection procedure and data collection tool. At the end of the second cycle, operating theatre staff were asked about the barriers to following the checklist using an interview sheet. This sheet had questions related to four themes, namely the need to follow the checklist, lack of feasibility, lack of awareness and training regarding the checklist, and lack of motivation. Statistical software program SPSS version 25.0 was used to analyze the data. Mean, and standard deviation was calculated for quantitative variables such as age. In addition, frequencies and percentages were calculated for categorical variables.

## 3 Results

We observed 23 operations in Audit Cycle 1, with the mean age of patients being 37.64 ± 18.62 years. 54.2% of our patients were females, and 45.2% were male. The patient board was filled in 26.1% of cases, and documentation of the surgical safety checklist in the patient file was done in 36.8% of cases. Sign-In was performed and read aloud in 65.2% and 13% of cases, respectively. Time out was performed and read aloud in 60.9% and 4.3%, respectively. Sign-out was performed and read aloud in 34.8% and 0%, respectively.

Sixteen operations were observed in Audit Cycle 2, with the mean age of patients was 41.84 ± 16.41 years. 43.8% of patients were female, and 56.3% were male. Improvement in compliance with the surgical safety checklist was significant after the educational intervention (Table 1).

**Table 1:** Improvement in compliance with the Surgical Safety Checklist.

	AUDIT CYCLE 1	AUDIT CYCLE 2
Patient Board Filled	26.1%	43.8%
Documentation	36.8%	62.5%
**SIGN IN**		
Performed	65.2%	81.3%
Read Aloud	13%	12.5%
**TIME OUT**		
Performed	60.9%	75%
Read Aloud	4.3%	6.1%
**SIGN OUT**		
Performed	34.8%	87.5%
Read Aloud	0%	6.3%

The greatest improvement in compliance (66.7%) was observed with the Sign-out step “Count of sponges and needles & instruments complete?” from 33.3% in Audit Cycle 1–100% in Audit Cycle 2. Whereas most minor improvement in compliance (4.2%) was observed with the Time-out step “Confirm all team members have introduced themselves by name & role?” from 8.3% to 12.5%.

Results of Audit Cycle 2 depicted a need to address barriers to compliance with the surgical safety checklist for significant self-sustaining improvement projects. Barriers were inquired from nurses, surgeons, anesthesiologists, and other OT staff via open-ended interview questions. 100% of respondents believed that “Staff is not aware and not trained to follow surgical safety checklist”. In addition, 81.1% believed that “No one initiates the process” whereas 62.5% of respondents thought that “There is no need to follow surgical safety checklist (SSC) as a nurse already confirms the required details before coming to operation theatre”. We categorize all the answers under four factors (Table 2).

**Table 2:** Barriers to compliance with the Surgical Safety Checklist.

**NO NEED TO FOLLOW SSC**	The nurse already confirms the required details of the patient before coming to the operation theatre.
	There is no need to read aloud components of the surgical safety checklist as we are already performing it.
	It doesn't help to improve morbidity or mortality.
**LACK OF FEASIBILITY**	Its time consuming and can't be followed in a chaotic OT environment.
	It can't be followed in an emergency as it causes a delay in surgical intervention.
	It increases the workload of OT staff.
	Sign-out can't be followed as surgeons have to take a rest before the subsequent surgery.
	It causes interruption to workflow during surgery.
	Members of the multidisciplinary team are at different locations during surgery, so it's not feasible to follow it.
**LACK OF AWARENESS AND TRAINING**	The staff is not aware and not trained to follow it.
**LACK OF MOTIVATION**	We are not provided with any incentive to follow it.
	No one initiates the process.
	I have not been assigned to follow it.

Based on these barriers, we felt that there is a need to make an appropriate intervention at the departmental level to make a sustainable quality improvement project for the future.

## 4 Discussion

The rationale of our quality improvement study was to assess the compliance of the WHO surgical safety checklist in our current surgical setting and devise a plan to efficiently follow it to improve perioperative patient safety and postoperative morbidity. The results of Audit Cycle 1 showed that there was better compliance with performance-related components of the checklist compared to verbal components. However, the checklist was also poorly documented in Cycle 1. Overall compliance to the checklist was improved in Audit Cycle 2 after educational intervention, but again, adherence to verbal components was poor compared to performance-related components.

Notably in our study, it was also found that the essential item of the checklist related to the mutual introduction of team members during the time-out stage was the least complied. It seems that operation theatre staff considered it unnecessary and time-consuming to follow the vocal parts of the checklist as they are already concentrating on performing the surgical procedure. A similar study in Sweden inferred the same result: those items of the checklist that promote communication among surgical staff were poorly applied, especially when introducing team members during the time-out stage. The reason may be the lack of awareness regarding safety associated with checklist implementation [12]. In addition, the scarcity of recognition concerning the purpose and benefits of the checklist among the operation room staff contributes to its poor conformity [13].

Our study showed that compliance improved during Audit cycle 2 after the educational intervention in the form of departmental presentation, which explains the positive association between them. This result is consistent with a study in which it is observed that the oral feedback to operation room staff and written feedback to the department encourage the use of the checklist [12]. Furthermore, in a prospective controlled intervention study, it was found in post-intervention surveys that compliance to the checklist was significantly improved with the resultant positive progress in patient safety and perception of staff related to the checklist [14]. The checklist essentially incorporates a coherent approach among the surgical team in terms of interpersonal communication and task completion, which certainly benefits the patient [3,15].

On inquiring to the operating staff about the possible hindrances in implementing the checklist, we came to know that the lack of awareness, training, motivation, and perception of insignificance about the checklist were the major barriers in the way of its application. This result is contrary to a study in England in which the resistance from senior faculty is the most common barrier to the checklist execution, followed by the lack of staff appropriate training and awareness [16]. Another study in France also deduced the opposite results. It appeared that the routinely pre-existing procedures in surgical settings cover some aspects of the checklist, which make the implementation of the checklist ineffective, and staff members, consider it the duplication of the checklist items. Other barriers were the communication gap among team members followed by the belief that the checklist is useless and too tedious to complete [17].

To the best of our knowledge, this is the first quality improvement study in our current setting, which makes an essential contribution to determining the compliance to the checklist and obstacles in the path of its accomplishment. Limitations include the lack of generalizability of the results as it focuses only on a surgical setting in one hospital. Moreover, it is short-term observational research. Therefore, the outcomes of this study do not reflect our country's contextual and departmental background. To get more clear and generalized results, multi-centred and long-term interventional studies are required in our country. These studies should involve the managerial, regulatory, and decision-making bodies such as the Department of Health that can directly influence the checklist implementation. From this, a clearer picture will be obtained to potentially fill the gaps in the process of the checklist execution [16]. On a small scale, as in our current setting, future replications of the audit will help to monitor the progress of the program.

As the surgical staff of our local surgical setting seriously lack communication during operative procedures, which can badly affect patient safety, Safety Attitude Questionnaires (SAQ) can be used to assess the quality and integrity of teamwork. On these bases, training programs can be initiated which will not only inculcate teamwork ethics and strengthen mutual communication among the team members, but also the verbal components of the checklist can be effectively followed during operating procedures [17,18]. To improve the compliance with the checklist at a departmental level, some measures can be applied, such as the recruitment of enthusiastic people from professional staff who can direct the surgical personnel during the operative procedures in some theatres to follow the checklist and compare the postoperative morbidity results with other theatres where such interference not done.

To overcome the barriers, evidence-based education and training that will clarify the checklist's crucial role in promoting interpersonal communication among team members and patients' health should be the priority. In addition, the checklist should be included in the patient healthcare delivery and replace the extra processes, which cause extra workload, with the checklist. Senior faculty members should use their leadership skills to help counter the resistance to implementing the checklist during surgical procedures by the junior staff. Moreover, local versions of the checklist and specifically subspeciality-oriented versions of the checklist should be developed for its smooth adoption [16,19].

## 5 Conclusion

From the results of our study, we can suggest that an important quality improvement tool i.e. WHO Surgical Safety Checklist, should be implemented and regularly followed in the surgical setting to facilitate the modification of surgical protocols which not only upgrade the patient safety measures but also integrate the teamwork skills and improve the local departmental culture.

## Ethical approval

Ethical approval for the audit was obtained from the respective surgical department.

## Source of funding

No funding required for the study.

## Author contribution

Fahad Gul and Maheen Nazir: Conception, design, write-up, implementation of audit, departmental audit presentation, critical review and approval of the final version. Khawar Abbas and Alishba Ashraf Khan: Conception, design, write-up, implementation of audit, critical review and approval of the final version. Daniya Shahzad Malick, Hashim Khan, Syed Naqash Haider Kazmi and Arbab Osama Naseem: Implementation of audit, critical review and approval of the final version.

## Trial register number

1.Name of the registry: Not applicable
2.Unique Identifying number or registration ID: Not applicable
3.Hyperlink to your specific registration (must be publicly accessible and will be checked): Not applicable

## Guarantor

Fahad Gul, Department of Surgery, Rawalpindi Medical University, Rawalpindi, Pakistan.

## Consent

Not required.

## Provenance and peer review

Not commissioned, externally peer-reviewed.

## Declaration of competing interest

All authors declared no conflict of interest.
